# Disease risk estimates in V30M variant transthyretin amyloidosis (A-ATTRv) from Mallorca

**DOI:** 10.1186/s13023-023-02865-5

**Published:** 2023-08-31

**Authors:** E. Cisneros-Barroso, F. Gorram, M. A. Ribot-Sansó, F. Alarcon, G. Nuel, J. González-Moreno, A. Rodríguez, J. Hernandez-Rodriguez, E. Amengual-Cladera, I. Martínez-López, T. Ripoll-Vera, I. Losada-López, D. Heine-Suñer, V. Plante-Bordeneuve

**Affiliations:** 1 Internal Medicine Department. Fundación Instituto de Investigación Sanitaria de Las Islas Baleares, Son Llàtzer University Hospital, Crta Manacor Km 4., 07198 Palma, Spain; 2 Balearic Research Group in Genetic Cardiopathies, Sudden Death and TTR Amyloidosis. Health Research Institute of the Balearic Islands (IdISBa), Son Llàtzer University Hospital, Palma, Spain; 3 Department of Neurology, University Hospital Henri Mondor, 51 Avenue du Maréchal de Lattre de Tasigny, 94000 Créteil, France; 4 Paris Est-Créteil University, Créteil, France; 5 Inserm U.955, Institut Mondor de Recherche Biomédicale (IMRB), Créteil, France; 6 Laboratory MAP5 UMR CNRS 8145, Paris University, Paris, France; 7 Stochastics and Biology Group, Department of Probability and Statistics (LPSM, UMR CNRS 8001), Sorbonne University, Paris, France; 8 Genomics of Health Research Group, Health Research Institute of the Balearic Islands (IdISBa), 07120 Palma, Spain; 9 Molecular Diagnostics and Clinical Genetics Unit, Hospital Universitario Son Espases, 07120 Palma, Spain; 10 Cardiology Department, Son Llàtzer University Hospital, Palma, Spain

**Keywords:** Transthyretin amyloidosis, Polyneuropathy, Genetics, Disease risk, Gene carriers

## Abstract

**Background:**

Variant transthyretin amyloidosis (A-ATTRv) is an autosomal dominant disease caused by a range of TTR gene variants which entail great phenotypical heterogeneity and penetrance. In Majorca, the A-ATTRv caused by the V30M gene variant (A-ATTRV30M) is the most common. Since asymptomatic carriers are at risk of developing the disease, estimating age of onset is vital for proper management and follow-up. Thus, the aim of this study was to estimate age-related penetrance in ATTRV30M variant carriers from Majorca.

**Methods:**

The disease risk among carriers from ATTRV30M families from Majorca was estimated by Non-parametric survival estimation. Factors potentially involved in the disease expression, namely gender and parent of origin were also analysed.

**Results:**

A total of 48 heterozygous ATTRV30M families (147 affected patients and 123 were asymptomatic carriers) were included in the analysis. Penetrance progressively increased from 6% at 30 years to 75% at 90 years of age. In contrast to other European populations, we observe a similar risk for both males and females, and no difference of risk according to the parent of origin.

**Conclusions:**

In this first study assessing the age-related penetrance of ATTRV30M variant in Majorcan families, no effect of gender or parent of origin was observed. These findings will be helpful for improving management and follow-up of TTR variant carrier individuals.

## Introduction

Amyloidosis refers to a group of diseases arising from the aggregation of structurally modified proteins, resulting in the formation of insoluble fibrils and deposition within various tissues and giving rise to organ complications. Currently, a total of 42 protein precursors linked to amyloidosis have been pinpointed, with Transthyretin (ATTR) standing out among them [1]. Transthyretin amyloidosis (A-ATTR) is caused either by variants in the *TTR* gene (A-ATTRv) or by the aggregation of wild-type TTR protein (A-ATTRwt) [2]. Variant transthyretin amyloidosis (A-ATTRv) is an autosomal dominant disease characterized by transthyretin-derived amyloid accumulation in various organs and tissues that leads to progressive dysfunction and eventually death [3]. More than 140 pathogenic variants of the *TTR* gene have been described so far, being V30M the most frequent worldwide. In the island of Majorca, A-ATTRV30M phenotype is considered endemic due to the high incidence of the disease [4–6].

A-ATTRv shows high phenotypic heterogeneity, with clinical manifestations including progressive peripheral (sensorimotor) and autonomic polyneuropathy, cardiomyopathy, nephropathy and gastrointestinal or ocular manifestations [3, 7]. The phenotype depends on the genetic variant, on the age of disease onset (AO) (which varies widely among different populations), and also on other factors not yet elucidated. This high heterogeneity makes A-ATTRv a challenging disease to recognize and manage, causing frequent diagnosis delays and misdiagnosis [3, 8]. Since currently available disease-modifying therapies can preserve the neurological function and reduce the disease burden [9], early diagnosis (and treatment) is of paramount importance to avoid significant and irreversible deterioration.

Once the A-ATTRv diagnosis is confirmed, presymptomatic genetic testing of relatives can help identifying asymptomatic carriers of *TTR* variants, who are at risk of developing A-ATTR. Establishing the risk these individuals have of developing A-ATTR (penetrance) is of utmost interest. Incomplete penetrance in variantcarriers has been previously described in different populations [10–14]. In addition, anticipation (AC), i.e., the occurrence of a significantly earlier AO within each generation has been described for A-ATTRV30M patients worldwide [15–18]. Finally, factors such as male gender or parent of origin (POO) effect (maternal) have also been proposed as risk factors for an enhanced penetrance [11, 13, 19, 20].

Current estimates of disease penetrance in variant carriers vary widely between populations and may be biased by uncertainties about the genetic variants. In our study, the aim was to provide reliable estimates of the disease risk in ATTRV30M Majorcan carriers. We searched a large sample of ATTRV30M families from Majorca and we evaluated potential factors modifying the AO through non-parametric survival estimation (NPSE).

## Materials and methods

Between 2002 and 2018, data from ATTRV30M families monitored at the multidisciplinary amyloidosis unit at the Hospital Universitario Son Llàtzer (Majorca) were collected. Based on the literature and on clinical experience, only adult subjects were included in the study.

For pedigree construction, raw data was anonymized, coded and entered on Pedigree XP software (version 2.1.091, 2015).

Demographics and clinical data were collected through personal communication with the index patient, their relatives and from the medical records. Demographics and clinical variables included were: age, gender, year of birth, date of last news (or death) for all individuals, and genotypic status, when available. The AO was set at the time of the first clinical manifestation related to the disease with certainty, as described elsewhere [21]. Patients with < 50 years at disease onset were classified as ‘early-onset’, and patients with ≥ 50 years at disease onset were classified as ‘late-onset’ patients.

### Statistical methods

Demographics and clinical variables were expressed as frequencies, percentages, means, standard deviations (SD) and range (min–max).

Disease risk estimate was determined using NPSE, as previously described [22]. For estimating the disease risk according to the POO, the algorithm calculated the likelihood of the transmitting parent depending on the whole structure of the family, as described elsewhere [23].

Differences of the penetrance curves between groups were evaluated using a likelihood ratio test in the Cox model. All analyses were conducted using R software (version 3.6.1) and survival package (version 2.37–4). Differences between groups for quantitative variables were compared using the Student’s t test, and for categorical variables with a chi–square test.

## Results

### Characteristics of A-ATTRv in Mallorca population

A total of 51 heterozygous ATTRV30M families were included in the study. Among them, 3 kindred were excluded due to lack of information, and eventually 48 families (428 subjects) were retained for analysis. The characteristics of the subjects included in the study are presented in Table 1. The present study included 147 A-ATTRv affected patientsand 123 asymptomatic carriers. Overall, there was a significant higher proportion of men, sex ratio 1.4 (*P* = 0.03). Besides, there was a higher proportion of males (58.5%) among affected patients, and a higher proportion of females among asymptomatic ATTRV30M carriers (56.9%). The mean AO was 50 ± 17 years, with a range from 22 to 82 years old. No differences on AO according to patients’ gender were observed. Early- and late-onset patients were observed simultaneously in 40% of families.

**Table 1:** Characteristics of the ATTRV30M amyloidosis families

	Total, n	Male, n	Female, n
Number of families	48	–	–
Studied subjects	428	250	178
Affected patients	147	86	61
Asymptomatic carriers	123	53	70
Mean AO ± SD [Range]	50 ± 17 [22–82]	47 ± 17 [22–82]	50 ± 15 [22–81]

### Penetrance profile

**Fig. 1:**
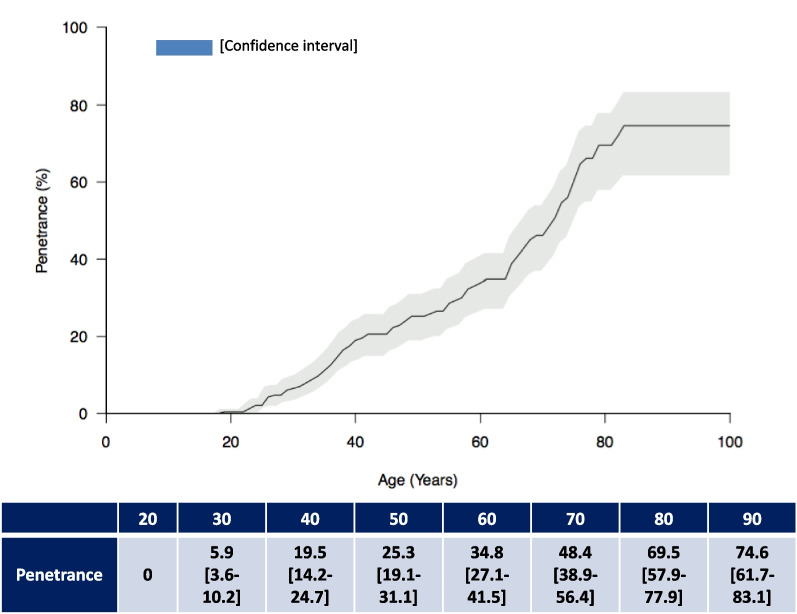
The disease risks (penetrance) in ATTRV30M families according to age

The age-related penetrance profile is shown in Fig. 1. The risk of disease increases gradually from the age of 25, where the penetrance is 4% [95% CI 1–6], to 25.3% [95% CI 19.1–31.1] and 48.4% [95% CI 38.9–56.4], at 50 and 70 years, respectively. Penetrance reached 69.5% [95% CI 57.9–77.9] at 80 years and remained stable from 80 years old onwards (Table 2).

**Table 2:** Disease risk estimates according to the age

Age (years)	Penetrance (in %) [95%CI]
20	0
30	5.9 [3.6–10.2]
40	19.5 [14.2–24.7]
50	25.3 [19.1–31.1]
60	34.8 [27.1–41.5]
70	48.4 [38.9–56.4]
80	69.5 [57.9–77.9]
90	74.6 [61.7–83.1]

**Fig. 2:**
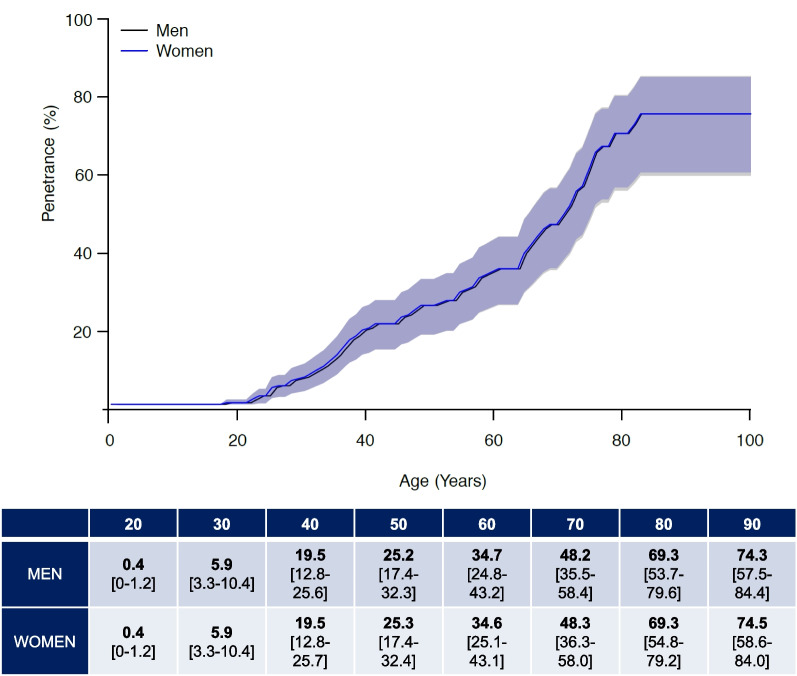
Disease risk estimates according to the gender

Male gender has been proposed as a potential risk factor for enhanced penetrance; therefore, we considered the disease risk by gender. We observed a similar risk for both male and female without significant differences (*P* = 0.91) (Fig. 2). Moreover, disease risk is stable from age 80 years probably because there is a lack of information in this age interval (Table 3).

**Table 3:** Disease risk estimates according to the gender and parent of origin

Age (years)	Gender* Penetrance (in %) [95%CI]		POO** Penetrance (in %) [95%CI]	
	Male (n = 234)	Female (n = 194)	Father	Mother
20	0.4 [0–1.2]	0.4 [0–1.2]	0.4 [0–1.8]	0.4 [0–1.2]
30	5.9 [3.3–10.4]	5.9 [3.3–10.4]	6.8 [3.2–10.2]	7.0 [3.3–10.5]
40	19.5 [12.8–25.6]	19.5 [12.8–25.7]	19.2 [12.6–25.3]	19.7 [13.0–25.9]
50	25.2 [17.4–32.3]	25.3 [17.4–32.4]	24.8 [17.0–31.9]	25.5 [17.7–32.6]
60	34.7 [24.8–43.2]	34.6 [25.1–43.1]	34.2 [24.3–42.7]	35.0 [25.4–43.4]
70	48.2 [35.5–58.4]	48.3 [36.3–58.0]	47.6 [34.9–57.8]	48.7 [36.7–58.4]
80	69.3 [53.7–79.6]	69.3 [54.8–79.2]	68.6 [52.9–79.0]	69.7 [55.2–79.5]
90	74.3 [57.5–84.4]	74.5 [58.6–84.0]	73.6 [56.7–83.9]	74.7 [59.1–84.3]

POO: parent of origin

*P value = 0.02; **P value < 0.001

**Fig. 3:**
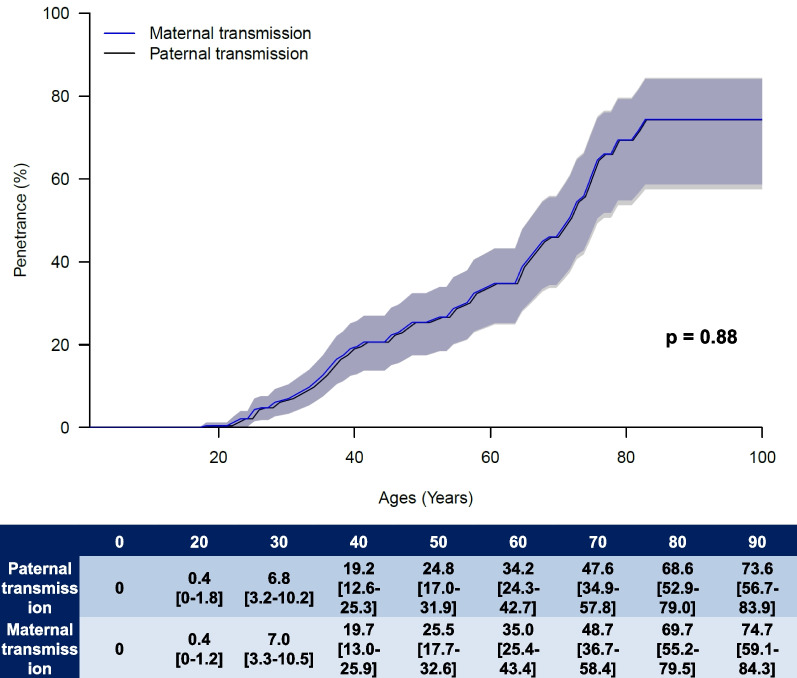
Disease risk estimates according to the parent of origin

Analysis of the disease risk according to the POO is shown in Fig. 3. No difference of risk according to the POO was observed (p = 0.88) (Fig. 3, Table 3).

## Discussion

The results from the present study provide data on the age-dependent penetrance profile of ATTRV30M variant carriers in Majorcan and factors potentially involved on gene penetrance.

The mean age of disease onset varies considerably among European A-ATTRV30M foci, ranging from the 30s in the Portuguese population, to the 60s in the Swedish population [14, 24, 25]. In the Majorcan population, a mean AO of 50 years was observed, in line with previously published data [26]. Thus, AO in Majorcan cohort may be considered an ‘intermediate’ value, with also a wide range of AO (22–82 years old). This broad range is also observed in both subpopulations with a similar mean AO [9] or higher/lower AO [13, 19, 25]. Besides, we observed simultaneous occurrence of early- and late-onset cases in 40% of the families. These ratios are in line with data reported in Sweden (30%) [13], but much higher than those observed in Portuguese and French kindred (12% and 15%, respectively) [25]. In the Majorcan cohort, a decrease in AO within each generation has been observed [27] and it may partially explain the coexistence of early and late onset populations.

Estimation of the disease risk is of outmost importance for asymptomatic carriers, and little data is available so far. When compared to other European population, the estimated disease risk was similar to French ATTRV30M families [10, 28]; it was lower than Portuguese population (with 95% at 90 years) [25], and it was higher than in Swedish population (with 64% at 80 years) [13, 25]. In addition, the risk increased steadily from the age of 25 years until the age of 85 years, with similar figures of sustained increase observed in other European ATTRV30M foci, particularly in Sweden and in France [10, 13]. Furthermore, penetrance was also observed to differ widely among patients carrying other variants, as for example, Val122Ile and Thr60Ala variants showing very low penetrance [25, 29–31].

The reported variability in penetrance, mean AO and AO range illustrates the high heterogeneity of the disease phenotype, which is probably linked to a multifactorial origin. Although not yet elucidated, several elements such as genetic [32], epigenetic [33] and environmental factors [34] have been proposed to explain this variation. One plausible hypothesis for the unique characteristics of our population is that 93% of subjects carrying V30M are also carriers of the G6S variant (unpublished data). G6S is considered a benign variant [35], but a role as a phenotype modifier cannot be discarded. Given that ATTRV30M foci do not share a geographic area, environmental factors should play a crucial role.

Previously published results in the Portuguese and Swedish ATTRV30M families [11, 13] showed higher penetrance and anticipation in case of maternal transmission. While we did not observe a POO effect on penetrance, we previously reported a trend towards an increased anticipation among offspring of affected mothers [27]. To note, the small size of our cohort could explain the lack of statistical difference based on the sex of affected parents.

### Strengths and limitations

This is the first study estimating disease risks on a large number of kindred from Majorcan families using a non parametric approach (NPSE) to optimize risks estimates [22]. This methodology does not require statistical law; it can manage all genotypes, and it also takes into account the covariates effect on the disease risk. Thus, it provided robust data useful for an age-dependent genetic disease such as A-ATTRv. Moreover, the NPSE method is designed for autosomal dominant transmission and its use has been validated in A-ATTRv [22].

Nevertheless, we are aware of some limitations and we assume that results must be interpreted in this context. In families from Majorca Island, one might expect consanguineous links; however, no homozygotic subjects have been reported in our population. Compared to other Spanish areas, Majorca Island do not show different runs of homozygosity, so the consanguinity would not be a major factor impacting on the present results [36].Thus, the sample included in this study is quite large and provides reliable data contributing to the existing body of evidence in the Majorcan ATTRV30M population.

### Clinical implications and further research

Currently, we have no tools to predict the disease AO or its penetrance, and no biomarkers for the disease onset have been identified so far. Therefore, all carriers must be strictly monitored from their identification onwards. Recently, new recommendations point out to start monitoring individuals at risk about 10 years prior to predicted AO [21]. In the future, identification of factors potentially involved in penetrance modulation, such as patients’ gender or environmental factors may help elucidating disease risk in future studies. Thus, further research focused on biomarkers and factors impacting on penetrance will be crucial to improve disease management and follow-up.

In conclusion, the results of the present study will be helpful to adjust genetic counselling and to improve management and follow-up of gene carrier individuals.

## Data Availability

The datasets used and/or analysed during the current study are available from the corresponding author on reasonable request.

## References

[1] Buxbaum JN, Dispenzieri A, Eisenberg DS, Fändrich M, Merlini G, Saraiva MJM (2022). Amyloid nomenclature 2022: update, novel proteins, and recommendations by the International Society of Amyloidosis (ISA) Nomenclature Committee. Amyloid.

[2] Rowczenio DM, Noor I, Gillmore JD, Lachmann HJ, Whelan C, Hawkins PN (2014). Online registry for mutations in hereditary amyloidosis including nomenclature recommendations. Hum Mutat.

[3] Ando Y, Coelho T, Berk JL, Cruz MW, Ericzon B-G, Ikeda S (2013). Guideline of transthyretin-related hereditary amyloidosis for clinicians. Orphanet J Rare Dis.

[4] Munar-Qués M, Saraiva MJM, Viader-Farré C, Zabay-Becerril JM, Mulet-Ferrer J (2005). Genetic epidemiology of familial amyloid polyneuropathy in the Balearic Islands (Spain). Amyloid.

[5] Munar-Ques M, Costa PP, Saraiva MJM, Viader-Farré C, Munar-Bernat C, Cifuentes-Luna C (1997). Familial amyloidotic polyneuropathy. TTR Met 30 in Majorca (Spain). Amyloid.

[6] Reinés JB, Vera TR, Martín MU, Serra HA, Campins MMC, Millán JMD (2014). Epidemiology of transthyretin-associated familial amyloid polyneuropathy in the Majorcan area: Son Llàtzer Hospital descriptive study. Orphanet J Rare Dis.

[7] Plante-Bordeneuve V (2018). Transthyretin familial amyloid polyneuropathy: an update. J Neurol.

[8] Cortese A, Vegezzi E, Lozza A, Alfonsi E, Montini A, Moglia A (2017). Diagnostic challenges in hereditary transthyretin amyloidosis with polyneuropathy: avoiding misdiagnosis of a treatable hereditary neuropathy. J Neurol Neurosurg Psychiatry.

[9] Koike H, Katsuno M (2020). Transthyretin amyloidosis: update on the clinical spectrum, pathogenesis, and disease-modifying therapies. Neurol Ther.

[10] Planté-Bordeneuve V, Carayol J, Ferreira A, Adams D, Clerget-Darpoux F, Misrahi M (2003). Genetic study of transthyretin amyloid neuropathies: carrier risks among French and Portuguese families. J Med Genet.

[11] Hellman U, Alarcon F, Lundgren H-E, Suhr OB, Bonaiti-Pellié C, Planté-Bordeneuve V (2008). Heterogeneity of penetrance in familial amyloid polyneuropathy, ATTR Val30Met, in the Swedish population. Amyloid.

[12] Saporta MAC, Zaros C, Cruz MW, André C, Misrahi M, Bonaïti-Pellié C (2009). Penetrance estimation of TTR familial amyloid polyneuropathy (type I) in Brazilian families. Eur J Neurol.

[13] Gorram F, Olsson M, Alarcon F, Nuel G, Anan I, Planté-Bordeneuve V (2021). New data on the genetic profile and penetrance of hereditary Val30Met transthyretin amyloidosis in Sweden. Amyloid Int J Exp Clin Investig Off J Int Soc Amyloidosis.

[14] Inês M, Coelho T, Conceição I, Duarte-Ramos F, de Carvalho M, Costa J (2018). Epidemiology of transthyretin familial amyloid polyneuropathy in portugal: a nationwide study. Neuroepidemiology.

[15] Lemos C, Coelho T, Alves-Ferreira M, Martins-da-Silva A, Sequeiros J, Mendonça D (2014). Overcoming artefact: anticipation in 284 Portuguese kindreds with familial amyloid polyneuropathy (FAP) ATTRV30M. J Neurol Neurosurg Psychiatry.

[16] Fraser FC (1997). Trinucleotide repeats not the only cause of anticipation. Lancet.

[17] Drugge U, Andersson R, Chizari F, Danielsson M, Holmgren G, Sandgren O (1993). Familial amyloidotic polyneuropathy in Sweden: a pedigree analysis. J Med Genet.

[18] Tashima K, Ando Y, Tanaka Y, Uchino M, Ando M (1995). Change in the age of onset in patients with familial amyloidotic polyneuropathy type I. Intern Med.

[19] Sousa A, Coelho T, Barros J, Sequeiros J (1995). Genetic epidemiology of familial amyloidotic polyneuropathy (FAP)-type I in Póvoa do Varzim and Vila do Conde (north of Portugal). Am J Med Genet.

[20] Bonaïti B, Alarcon F, Bonaïti-Pellié C, Planté-Bordeneuve V (2009). Parent-of-origin effect in transthyretin related amyloid polyneuropathy. Amyloid.

[21] Conceição I, Damy T, Romero M, Galán L, Attarian S, Luigetti M (2019). Early diagnosis of ATTR amyloidosis through targeted follow-up of identified carriers of TTR gene mutations. Amyloid Int J Exp Clin Investig Off J Int Soc Amyloidosis.

[22] Alarcon F, Planté-Bordeneuve V, Olsson M, Nuel G (2018). Non-parametric estimation of survival in age-dependent genetic disease and application to the transthyretin-related hereditary amyloidosis. PLoS ONE.

[23] Alarcon F, Planté-Bordeneuve V, Nuel G. Study of the Parent-of-origin effect in monogenic diseases with variable age of onset. Appl ATTRv. 2023 10.1371/journal.pone.0288958 PMC10414668 37561731

[24] Olsson M, Jonasson J, Cederquist K, Suhr OB (2014). Frequency of the transthyretin Val30Met mutation in the northern Swedish population. Amyloid Int J Exp Clin Investig Off J Int Soc Amyloidosis.

[25] Planté-Bordeneuve V, Gorram F, Olsson M, Anan I, Mazzeo A, Gentile L, et al. A multicentric study of the disease risks and first manifestations in Hereditary transthyretin amyloidosis (ATTRv): insights for an earlier diagnosis. Amyloid. 2023;1–8. 10.1080/13506129.2023.2178891 36994840

[26] Buades-Reinés J, Raya-Cruz M, Gallego-Lezaún C, Ripoll-Vera T, Usón-Martín M, Andreu-Serra H (2016). Transthyretin familial amyloid polyneuropathy (TTR-FAP) in Mallorca: a comparison between late- and early-onset disease. J Peripher Nerv Syst.

[27] Cisneros-Barroso E, González-Moreno J, Rodríguez A, Ripoll-Vera T, Álvarez J, Usón M (2020). Anticipation on age at onset in kindreds with hereditary ATTRV30M amyloidosis from the Majorcan cluster. Amyloid Int J Exp Clin Investig Off J Int Soc Amyloidosis.

[28] Gorram F, Alarcon F, Perdry H, Hébrard B, Damy T, Fanen P (2017). Refine penetrance estimates in the main pathogenic variants of transthyretin hereditary (familial) amyloid polyneuropathy (TTR-FAP) using a new non-parametric approach (NPSE). Amyloid Int J Exp Clin Investig Off J Int Soc Amyloidosis.

[29] Jacobson DR, Pastore RD, Yaghoubian R, Kane I, Gallo G, Buck FS (1997). Variant-sequence transthyretin (isoleucine 122) in late-onset cardiac amyloidosis in black Americans. N Engl J Med.

[30] Reilly MM, Staunton H, Harding AE (1995). Familial amyloid polyneuropathy (TTR ala 60) in north west Ireland: a clinical, genetic, and epidemiological study. J Neurol Neurosurg Psychiatry.

[31] Wechalekar AD, Gillmore JD, Hawkins PN (2016). Systemic amyloidosis. Lancet.

[32] Dias A, Santos D, Coelho T, Alves-Ferreira M, Sequeiros J, Alonso I (2019). C1QA and C1QC modify age-at-onset in familial amyloid polyneuropathy patients. Ann Clin Transl Neurol.

[33] De Lillo A, Pathak GA, De Angelis F, Di Girolamo M, Luigetti M, Sabatelli M (2020). Epigenetic profiling of Italian patients identified methylation sites associated with hereditary transthyretin amyloidosis. Clin Epigenetics.

[34] Hardell L, Holmgren G, Steen L, Fredrikson M, Axelson O (1995). Occupational and other risk factors for clinically overt familial amyloid polyneuropathy. Epidemiology.

[35] Sikora JL, Logue MW, Chan GG, Spencer BH, Prokaeva TB, Baldwin CT (2015). Genetic variation of the transthyretin gene in wild-type transthyretin amyloidosis (ATTRwt). Hum Genet.

[36] Biagini SA, Solé-Morata N, Matisoo-Smith E, Zalloua P, Comas D, Calafell F (2019). People from Ibiza: an unexpected isolate in the Western Mediterranean. Eur J Hum Genet England.

